# Preventing autoimmune arthritis using antigen-specific immature dendritic cells: a novel tolerogenic vaccine

**DOI:** 10.1186/ar2031

**Published:** 2006-08-15

**Authors:** Igor Popov, Mu Li, Xiufen Zheng, Hongtao San, Xusheng Zhang, Thomas E Ichim, Motohiko Suzuki, Biao Feng, Costin Vladau, Robert Zhong, Bertha Garcia, Gill Strejan, Robert D Inman, Wei-Ping Min

**Affiliations:** 1 Department of Surgery, Microbiology and Immunology, and Pathology, London Health Science Centre, London, Canada; 2 Multi-Organ Transplant Program, London Health Science Centre, London, Canada; 3 Immunology and Transplantation, Lawson Health Research Institute, London, Canada; 4 Robarts Research Institute, London, Canada; 5 Division of Rheumatology, Department of Medicine, Toronto Western Hospital, University Health Network, Toronto, Canada

## Abstract

Conventional treatments for autoimmune diseases have relied heavily on nonspecific immune suppressants, which possess a variety of adverse effects without inhibiting the autoimmune process in a specific manner. In the present study we demonstrate the effectiveness of antigen-specific, maturation-resistant, tolerogenic dendritic cells (DC) in suppressing collagen-induced arthritis, a murine model of rheumatoid arthritis. Treatment of DC progenitors with the NF-κB inhibiting agent LF 15-0195 (LF) resulted in a population of tolerogenic DC that are characterized by low expression of MHC class II, CD40, and CD86 molecules, as well as by poor allostimulatory capacity in a mixed leukocyte reaction. Administering LF-treated DC pulsed with keyhole limpet hemocyanin antigen to naïve mice resulted hyporesponsiveness specific for this antigen. Furthermore, administration of LF-treated DC to mice with collagen-induced arthritis resulted in an improved clinical score, in an inhibited antigen-specific T-cell response, and in reduced antibody response to the collagen. The efficacy of LF-treated DC in preventing arthritis was substantiated by histological examination, which revealed a significant decrease in inflammatory cell infiltration in the joints. In conclusion, we demonstrate that *in vitro*-generated antigen-specific immature DC may have important potential as a tolerogenic vaccine for the treatment of autoimmune arthritis.

## Introduction

The natural function of immature dendritic cells (DC) is to provide conditions for self-tolerance, either through the generation of regulatory T cells or through the induction of apoptosis or anergy of autoreactive effector cells [1-3]. Several attempts have been made to utilize immature DC therapeutically. Some hurdles unfortunately still exist that prevent the therapeutic use of immature DC: first, only limited protocols are available for generating immature DC; and second, there is a danger that once immature DC are introduced into the host, a maturation event may occur that would actually cause immunogenicity instead of tolerance [4,5]. A direct method of targeting DC maturation involves blocking signal transduction pathways that are necessary for the DC to differentiate. A pathway known to be involved in DC maturation is the cascade that leads to activation of the transcription factor NF-κB. Zanetti and colleagues established that the RelB component of NF-κB is critical for DC maturation *in vivo *[6].

LF 15-0195 (LF) is a chemically synthesized analog of the immune suppressant 15-deoxyspergualin that possesses higher immunosuppressive activity and less *in vivo *degradation than its parent compound [7]. It has been demonstrated that part of the immune suppressive effects of LF are due to activation of caspases in reactive T cells [8].

Our laboratory has focused on the antigen-presenting cell arm of the immune system. We have been the first to demonstrate that LF specifically interferes with DC maturation through inhibiting the activity of IκB kinase (IKK) on its target IKB [9]. The unique ability of LF to target IKK in DC therefore suggests that it may possess distinctive properties allowing the generation of immature tolerogenic DC. Supporting the role of LF as a tolerogenic agent are studies describing induction of 'active' long-term tolerance in situations of autoimmunity, as illustrated in models of experimental autoimmune encephalomyelitis [10,11] and of myasthenia gravis [12].

Our group has also successfully induced tolerance in transplantation by LF treatment [13]. LF had a significant cytotoxic impact *in vivo*, however, thus emphasizing the possible deleterious effects of LF therapy [7]. To avoid such negative side effects, we chose to generate Tol-DC in *vitro *by treatment with LF, which may represent a safer, more natural, and potentially clinically applicable alternative to LF systemic administration.

Rheumatoid arthritis (RA) is an autoimmune disease that selectively targets joint tissue, causing significant disability and loss of function. Although we have previously demonstrated that systemic LF treatment combined with T-cell modulation can selectively expand tolerogenic DC in a transplantation model [14], the ability of tolerogenic DC generated *in vitro *to serve as an antigen-specific tolerogenic tool has not been shown. Stimulated by the possibility of combining the immunosuppressant properties of LF and the therapeutic potential of DC, we sought to generate antigen-specific Tol-DC *in vitro *using LF, and to use these cells as therapeutic tools to inhibit RA.

In the present study, we evaluated the ability of LF to generate a population of Tol-DC. Using collagen-induced arthritis (CIA), a murine model of RA, we show that LF-treated DC when pulsed with antigen and adoptively transferred into naïve syngeneic recipients selectively induce hyporesponsiveness at the level of both T cells and B cells. We further investigated whether such LF-treated DC can be used in a therapeutic context in order to induce amelioration of ongoing arthritis pathology, and show that the treated mice exhibited decreased inflammatory cell infiltration in the joints. Taken together, these data indicate that LF-generated tolerogenic DC have a therapeutic role in the inhibition of CIA.

## Materials and methods

### Animals

Male DBA/1 LacJ mice and BALB/c mice (Jackson Laboratories, Bar Harbor, ME, USA) were kept in filter-top cages at the Animal Facility, University of Western Ontario according to National Canadian Council for Animal Guidelines. Mice were allowed to settle for 2 weeks before the initiation of experimentation, which had ethical approval from the university board.

### Collagen-induced arthritis model

DBA/1 mice, 7 weeks of age, were intradermally immunized at several sites into the base of the tail with 200 μg bovine type 2 collagen (CII) dissolved in 100 μl of 0.05 M acetic acid and mixed with an equal volume of complete Freund's adjuvant (Sigma, Oakville, ON, Canada). CII was dissolved at a concentration of 2 mg/ml by stirring overnight at 4°C. On day 21, the mice received an intraperitoneal booster injection with 200 μg CII in an equal volume (100 μl) of PBS. The booster injection was necessary to induce reproducible CIA, which normally developed at about day 28.

Each mouse was examined visually three times per week for the appearance of arthritis in limb joints, and the arthritis score was given as follows: 0, no detectable arthritis; 1, erythema and mild swelling confined to the mid-foot or ankle joint; 2, significant swelling and redness; 3, severe swelling and redness from the ankle to digits; and 4, maximal swelling and redness or obvious joint destruction associated with visible joint deformity or ankylosis. Each limb was graded and expressed as the average score per affected paw, resulting in a maximum score of 4 per animal. Scoring was performed by two independent observers, without knowledge of experimental protocols.

### Dendritic cell cultures

At day 0, bone marrow cells were flushed from the femurs and tibias of DBA/1 mice, and were washed and cultured in six-well plates (Corning, Acton, MA, USA) at 4 × 10^6 ^cells/well in 4 ml complete medium (RPMI 1640 supplemented with 2 mM L-glutamine, 100 U/ml penicillin, 100 μg streptomycin, 50 μM 2-ME, and 10% FCS (all from Invitrogen, Grand Island, NY, USA) supplemented with recombinant granulocyte-macrophage colony-stimulating factor (GM-CSF) (10 ng/ml) and recombinant mouse IL-4 (10 ng/ml) (both from PeproTech, Rocky Hill, NJ, USA). Cultures were incubated at 37°C in 5% humidified CO_2_.

Nonadherent cells were then removed (day 2) and fresh medium was added. At day 4 the DC were treated either with LF (5–10 μg/ml) or with PBS, and fresh medium was added every 24 hours. At day 7 we pulsed LF-treated DC or PBS-treated DC with CII (10 μg/ml) for 24 hours. DC were then activated with lipopolysaccharide (LPS) (10 ng/ml; Sigma) and tumor necrosis factor alpha (TNFα) (10 ng/ml; PeproTech) for an additional 24 hours, were washed extensively, and were used for subsequent transfer experiments. On day 12 after the CII priming, different groups of mice with four to six animals per group were injected intraperitoneally with these LF-treated DC or untreated DC (5 × 10^6 ^cells/mouse).

### Dendritic cell vaccination and antigen-specific response

In some experiments, day 4 bone marrow DC from BALB/c mice, cultured in GM-CSF/IL-4, were treated with LF (0.1, 1 or 10 μg/ml) or PBS, and fresh medium without LF was added every 24 hours. At day 7, we pulsed LF-treated or PBS-treated DC with keyhole limpet hemocyanin (KLH) (10 μg/ml) (Sigma) for 24 hours. DC were then activated with LPS/TNF-α for an additional 24 hours and injected subcutaneously (5 × 10^5 ^cells/mouse) into syngeneic mice. The mice were sacrificed after 10 days, and T lymphocytes from draining lymph nodes and spleens were isolated. Finally, a KLH-specific recall response was performed as described later.

### Mixed lymphocyte reaction

At day 4 of culture, bone marrow DC from DBA/1 LacJ mice were treated with LF (10 μg/ml) or PBS, followed by addition of LPS/TNFα at day 8 for 24 hours. Activated DC were irradiated (3,000 rad) and seeded in triplicate into a flat-bottom 96-well plate (Corning) as stimulators. Spleen T cells from BALB/c mice were isolated by gradient centrifugation over Ficoll-Paque (Amersham, Canada) and added as responders (5 × 10^5 ^cells/well). The mixed lymphocytes were cultured at 37°C for 72 hours in 200 μl RPMI 1640 supplemented with 10% FCS, 100 U/ml penicillin, and 100 μg/ml streptomycin, and were pulsed with 1 μCi/well [^3^H-labeled] thymidine (Amersham) for the last 16 hours of culture. Finally, cells were harvested onto glass fiber filters, and the radioactivity incorporated was quantitated using a Wallac Betaplate liquid scintillation counter (Beckman, Fullerton, CA, USA). Results were expressed as the mean counts per minute of triplicate cultures ± SEM.

### Proliferation assays

Proliferative responses to KLH and CII in subsequent groups of mice were measured with a standard microtiter assay using either draining lymph node cells or splenocytes, using KLH or CII, and using ^3^H-labeled thymidine. T cells at 5 × 10^5^/well were seeded into a 96-well flat-bottom microtiter plate in triplicate and mixed with serial dilutions of KLH or CII (5–50 μg/well). Following a 72-hour incubation, 1 μCi [^3^H] thymidine was added to each well for 16 hours. Using a cell harvester, the cells were collected onto a glass microfiber filter, and the radioactivity incorporated was measured by a Wallac Betaplate liquid scintillation counter.

### Anti-type II collagen antibody measurement

CII-specific antibodies were evaluated using a standard indirect ELISA in which 500 ng CII was absorbed to each well of a 96-well microtiter plate. Following blocking and washing steps, serial dilutions of immune mouse serum (1:100-1:100,000) were added to the appropriate wells in duplicate and were incubated overnight at 4°C. To develop the ELISA, horseradish peroxidase-conjugated goat anti-mouse IgG Fc and orthophenylenediamine dihydrochloride substrate buffer (Sigma) were used. Finally, the optical density into each well was measured at 490 nm wavelength in an ELISA plate reader.

### Cytokine quantification

LF-treated DC of DBA/1 origin were cultured alone or with the allogeneic (BALB/c) T cells for 48 hours. Supernatants were collected and assessed for DC (IL-10, IL-12) and for T-cell cytokines (interferon gamma, IL-4). An ELISA (Endogen, Rockford, IL, USA) was used for detecting cytokine concentrations in the supernatants according to the manufacturer's instructions using a Benchmark Microplate Reader (Bio-Rad, Hercules, CA, USA).

### Histology

Paws of freshly dissected mice were removed and joint tissues were immersion-fixed for 4 days in 10% (wt/vol) neutral buffered formalin in 0.15 M PBS (pH 7.4). After decalcification in Decalcifier I solution (Surgipath, Richmond, IL, USA) overnight and subsequent dehydration in a gradient of alcohols, tissues were rinsed in running water. The specimens were processed for paraffin embedding in paraplast (BDH, Dorset, UK) as routine procedure. Serial paraffin sections throughout the joint were cut at 5 μm thickness on a microtome, heated at 60°C for 30 minutes, and were deparaffinized. Hydration was achieved by transferring the sections through the following solutions: three times through xylene for 6 minutes, and then for 2 minutes through 100% ethanol twice, 95% ethanol, and 70% ethanol, respectively. The sections were stained with H & E and were mounted on glass slides.

### Flow cytometry

Phenotypic analysis of cells was performed using flow cytometry on a FACScan (Becton Dickinson, San Jose, CA, USA). DC were pretreated with LF (5-10 μg/ml) beginning at day 4. Activation of DC maturation was performed by addition of TNFα/LPS for 24 hours. The cells were stained with FITC-conjugated mAbs against surface markers associated with DC maturation (anti-mouse CD11c, I-A, CD40, and CD86; Cedarlane, Hornby, ON, Canada). Immunoglobulins of the same isotype were used as controls.

### Statistical analysis

Data are expressed as the mean ± SEM. Differences in the arthritis score between different populations of mice were compared using the Mann-Whitney U test for nonparametric data. *P *< 0.05 was considered significant.

## Results

### Modulation of dendritic cell maturation and function by LF 15-0195

**Figure 1:**
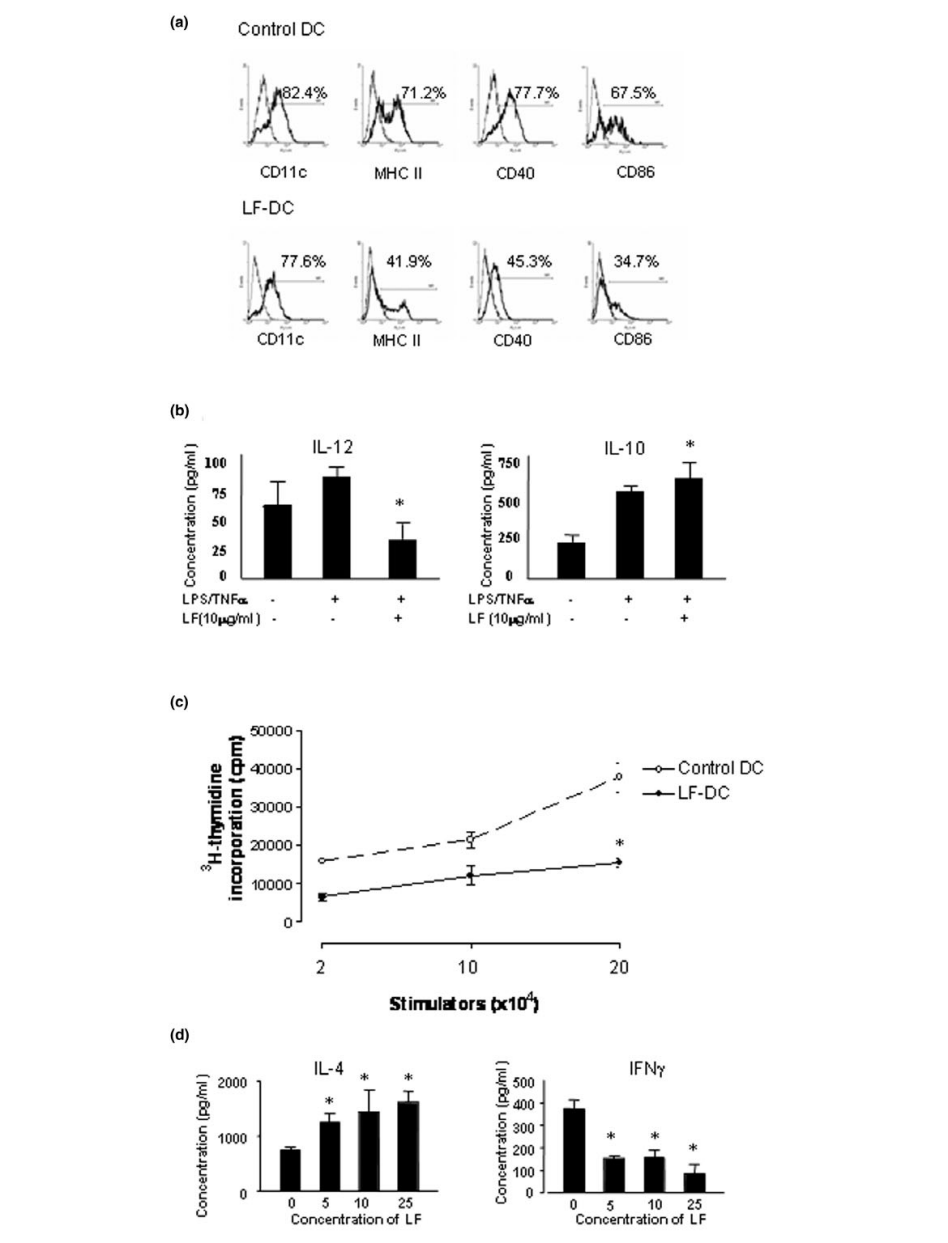
LF 15-0195 prevents maturation and function of dendritic cells. **(a) **Phenotypic analysis of LF-treated dendritic cells (DC). Bone-marrow-derived DC were cultured in the presence of granulocyte-macrophage colony-stimulating factor (10 ng/ml) and IL-4 (10 ng/ml) for 7 days. Control mature DC (upper panels) were activated using tumor necrosis factor alpha (TNFα)/lipopolysaccharide (LPS) in the last 24-hour culture. DC (lower panel) were treated by addition of LF (10 ng/ml) in the culture medium from day 4 onwards, and fresh medium was added every 24 hours. DC were stained with FITC-conjugated mAbs and analyzed by flow cytometry. Results represent one of three experiments (*n *= 4 per group/experiment). **(b) **LF regulates cytokine expression in DC. DC were treated with LF as in (a). The supernatants of DC culture were collected and used to measure IL-12 and IL-10 levels by ELISA as described in Materials and methods. *P < 0.05, comparing untreated control DC.**(c) **LF inhibits DC allostimulatory capacity in a mixed leukocyte reaction. DC were pretreated with LF and subsequently stimulated with 10 ng/ml TNFα/LPS as described in (a). DBA/1 control DC and LF-treated DC, at indicated concentrations, were used as stimulators, and BALB/c splenocytes (1 × 10^5^/well) were used as responders. Stimulators and responders were cocultured, and proliferation was assessed as described in Materials and methods. Data shown are representative of three independent experiments (*n *= 4 per group/experiment).*P *< 0.05, comparing untreated control DC.**(d) **LF-treated DC regulate T helper cell deviation. LF-treated DC and PBS-treated control DC (10^6^) (DBA/1) were subsequently cultured with allogeneic (BALB/c) T cells (10^7^) for 48 hours. Supernatants were collected from the cultures and interferon gamma (IFNγ; Th1) and IL-4 cytokine (Th2) levels were measured by ELISA. Results represent one of three experiments (*n *= 4 per group/experiment). *P *< 0.05, comparing untreated control DC.

Our previous studies have demonstrated that LF together with anti-CD45RB mAb can induce a population of tolerogenic DC in transplant recipients that are responsible for maintenance of tolerance [14]. Furthermore, we have previously demonstrated that LF treatment of isolated DC *in vitro* is capable of inhibiting the maturation-inducing kinase IKK, as well as the downstream transcription factor NF-κB [14]. We therefore investigated the potential of LF to generate immature tolerogenic DC that could be used for antigen-specific immunotherapy *in vivo*. Bone-marrow-derived DC were generated using a standard 7-day culture in GM-CSF/IL-4. LF was added at day 4 of culture, whereas control DC were treated with PBS alone. Activation of control DC and LF-treated DC was performed by addition of TNFα/LPS for 24 hours. Assessment of MHC class II, CD40, and CD86 expression by flow cytometry revealed that control DC underwent marked maturation, whereas LF-treated DC did not upregulate maturation markers (Figure 1a). Both nonactivated control DC and LF-treated DC expressed low levels of the maturation markers, similar to the TNFα/LPS-activated LF-treated DC (data not shown).

We next assessed whether LF is involved in regulation of DC cytokine expression. LF-treated DC following activation with LPS/TNFα were cultured alone for 48 hours. Supernatants were then used to measure levels of IL-12 and IL10 cytokines. As shown in Figure 1b, IL-12 production of LF-treated DC was reduced, whereas IL-10 production reciprocally upregulated.

Functional assessment of LF-treated DC was performed using these cells as allogeneic stimulators in a mixed lymphocyte reaction (MLR). In contrast to control-DC-expressed potent allostimulatory activity, LF-treated DC evoked a much weaker proliferative response (Figure 1c). Using LF-treated DC as stimulators of MLR resulted in preferential production by T cells of the Th2 cytokine IL-4 and reduction of the Th1 cytokine interferon gamma (Figure 1d), in contrast to stimulation with control DC. These data suggest that LF treatment can effectively endow DC with an immature phenotypic and functional state.

### LF 15-0195-treated dendritic cells inhibit an antigen-specific T-cell response

We next used LF-treated DC as a platform for the delivery of antigens in a tolerogenic context. It has previously been reported that antigen-pulsed DC with a blocked NF-κB pathway can induce specific hyporesponsiveness to that antigen [15]. Since we have recently demonstrated that LF blocks NF-κB translocation [9], and we have shown here that LF treatment inhibits DC maturation, we sought to assess whether LF-treated DC could induce tolerance to a nominal antigen such as KLH.

Pulsing of DC with antigen requires active cellular phagocytosis and processing of the antigen. The *in vivo *administration of the antigen-pulsed DC is subjected to conditions that may induce maturation not normally present *in vitro*. Since this is the first use of LF for treatment of DC before antigen pulsing, we performed optimization experiments to determine the most effective concentration of LF. On day 4 of culture, bone marrow DC were treated with 0.1, 1, and 10 μg/ml LF, and control DC were treated with PBS. KLH was added to DC at day 7 for 24 hours, and subsequently cells were activated with TNFα/LPS. On day 9, 5 × 10^5 ^DC were injected intraperitoneally into BALB/c mice.

**Figure 2:**
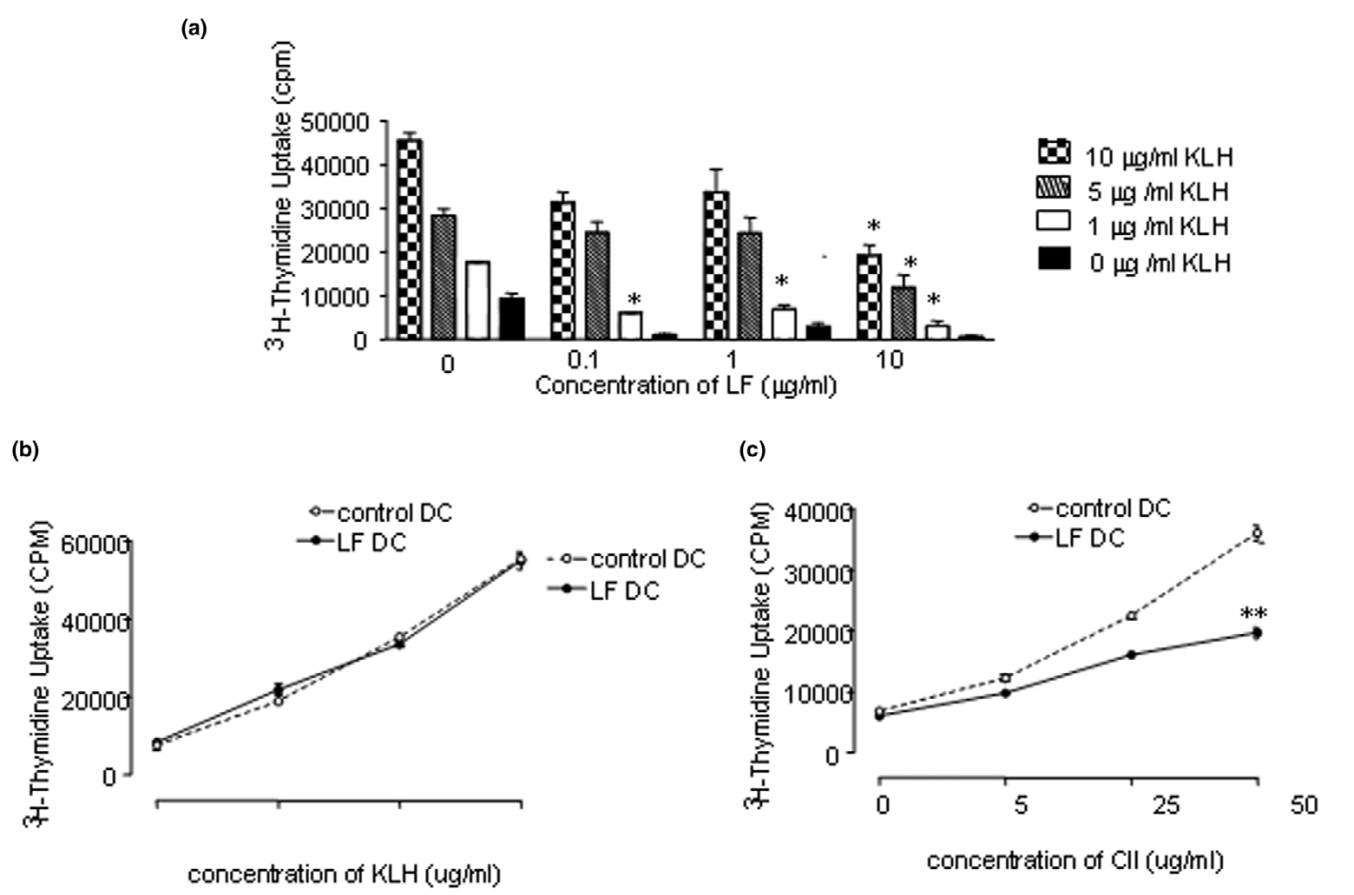
LF 15-0195-treated dendritic cells inhibit antigen-specific T-cell responses. **(a) **LF-treated dendritic cells (DC) inhibit anti-keyhole limpet hemocyanin (KLH) T-cell responses. Day 4 bone-marrow-derived DC cultured in granulocyte-macrophage colony-stimulating factor (10 ng/ml) and IL-4 (10 ng/ml) were treated with different concentrations of LF (0.1, 1, and 10 μg/ml) or PBS alone. On day 7 of culture, 10 μg/ml KLH was added to the cells for 24 hours and then cells were activated with TNFα (10 ng/ml) and lipopolysaccharide (10 ng/ml). On day 9 of DC culture, 5 × 10^5 ^cells/mouse were injected intraperitoneally into syngeneic BALB/c mice. After 10 days, the mice were sacrificed and T cells from lymph nodes were isolated. A KLH-specific recall response was determined by the proliferation, as described in Materials and methods. *P < 0.05 versus nontreated control DC. **(b) **and **(c) **LF-treated DC-induced immune suppression is antigen specific. DC were cultured, treated with LF, pulsed with type II collagen (CII) antigen, and immunized mice as described in (a). Two days prior to LF-treated DC or untreated control DC immunization, the mice were immunized with 10 μg KLH subcutaneously. Ten days after immunization, lymph node cells were harvested and proliferated *in vitro *in the presence of CII (b) and KLH (c), respectively, at the indicated concentrations. Results represent one of three experiments. *P < 0.05 versus nontreated control DC. cpm, counts per minute.

To test the T-cell expansion and activation, the recall response to KLH was assessed *in vitro *10 days after the administration of KLH-pulsed control DC and LF-treated DC. KLH-specific responses from lymph node T cells were suppressed at all KLH concentrations used, in an LF dose-dependent manner (Figure 2a). To determine whether bystander tolerization occurred in LF-treated DC-induced immune suppression, we used a 'double immunization' system, in which mice were immunized with CII-pulsed DC alone with an immunization with KLH. The immunization with LF-treated DC and CII antigen-pulsed DC only suppressed the immune response to CII specifically (Figure 2b), but not the immune response to the nonrelevant antigen KLH (Figure 2C).

### Inhibition of collagen-induced arthritis development by LF 15-0195-treated dendritic cells

The CIA model of arthritis is a well-established method of evaluating therapeutic interventions in autoimmune arthritis. Several induction protocols have been reported, all of which in essence induce a T-cell-dependent inflammatory infiltration of the synovial membrane, leading to cartilage destruction and bone erosion. Since we have been able to induce T-cell hyporesponsiveness to KLH using LF-treated DC (Figure 2), we sought to determine whether pulsing LF-treated DC with CII would inhibit CIA development and histopathology. On day 12 post CII priming, DBA/1 mice were administered 5 × 10^6 ^intraperitoneal CII-pulsed LF-treated DC or control DC. A booster injection of CII was made at day 21. The clinical onset of CIA as determined by the average arthritis score per effected paw began approximately on day 28.

**Figure 3:**
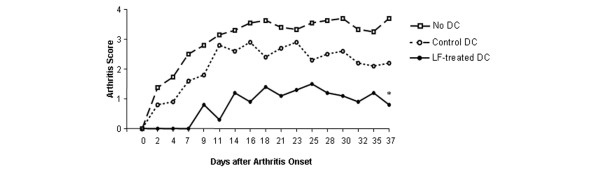
Type II collagen-pulsed LF 15-0195-treated dendritic cells inhibit clinical development of collagen-induced arthritis. Twelve days after intradermal challenge with type II collagen (CII) (200 μg/mouse in complete Freund's adjuvant), DBA/1 LacJ mice were injected intraperitoneally with LF-treated dendritic cells (DC) (5 μg/ml) and CII-pulsed DC (10 μg/ml) (5 × 10^6 ^cells/mouse). Controls were either treated with non-LF-treated but CII-pulsed DC or remained untreated. All mice were boosted by an intraperitoneal injection with the same dose of CII in PBS 9 days later. The mice were observed for 37 days after arthritis onset. Each limb was graded on a scale from 0 to 4 and the average clinical score per affected paw was calculated. Each point denotes the score of six mice in each group. Results represent one of three experiments. *P < 0.05 versus the control DC-treated group.

Initiation of arthritis was delayed by 7 days in the CII-pulsed LF-treated DC group as compared with the control group. Furthermore, the control group had an average score per affected paw twice as high as that of the LF-treated DC group (Figure 3), but a score that ranged from less than twofold to fivefold depending on the time point. These results imply that LF-treated DC are not only capable of inducing antigen-specific hyporesponsiveness, but are also capable of reducing clinical manifestations and delaying disease onset in a model of autoimmunity.

### Inhibition of collagen-induced arthritis is associated with long-term T-cell hyporesponsiveness

**Figure 4:**
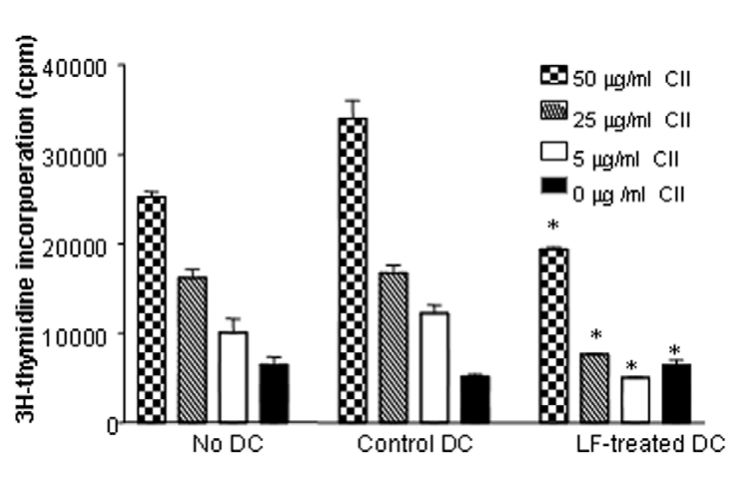
T-cell hyporesponsiveness to type II collagen in collagen-induced arthritis-susceptible mice with LF-treated dendritic cells. Day 4 bone-marrow-derived dendritic cells (DC) cultured in granulocyte-macrophage colony-stimulating factor/IL-4 were treated with 5 μg/ml LF or PBS alone, and fresh medium was added every 24 hours. On day 7, both LF-treated DC and control PBS-treated DC were pulsed with type II collagen (CII) (10 μg/ml) for 24 hours. On day 8, CII-pulsed cell cultures were activated with TNFα/lipopolysaccharide for the next 24 hours, and 5 × 10^6 ^cells/mouse were injected intraperitoneally into DBA/1 LacJ mice primed with CII (200 μg/mouse in complete Freund's adjuvant) 12 days earlier. Twenty-one days after priming, the mice were boosted intraperitoneally with the same dose of CII in PBS. At the end of clinical assessment of collagen-induced arthritis development, the mice were sacrificed and T cells from lymph nodes were isolated. A CII-specific response from different groups of animals was performed by proliferation, as described in Materials and methods. Lymphocytes were restimulated *in vitro *with different concentrations of CII (5, 25, and 50 μg/ml) or PBS alone and a ^3^H-labeled thymidine incorporation was measured. Results represent one of three experiments (*n *= 4 per group/experiment). *P < 0.05 versus the control DC-treated group. cpm, counts per minute.

Given that T cells play a key role in the initiation of CIA [16], antigen-specific T-cell proliferative responses to CII were assessed. At the end of the monitoring of CIA development, mice were sacrificed and lymph node cells were collected for proliferative analysis in response to CII. *In vitro *^3^H-labeled thymidine incorporation assays revealed that a decrease in CII-specific recall responses were observed of mice receiving LF-treated DC in comparison with those receiving control DC (Figure 4). The response was antigen specific since modulation of responses to other control antigens was not affected (data not shown). The hyporesponsiveness of CII-specific T cells confirms clinical observations that CII-pulsed LF-treated DC could be useful in therapeutic intervention for antigen-specific T-cell-associated diseases.

### Inhibition of collagen-induced arthritis is also associated with prolonged inhibition of anti-type II collagen antibodies

The importance of antibodies in development of CIA pathology is well known [17]. Although it has been previously suggested that LF directly inhibits antibody production [18], the ability of the LF-treated DC to induce this effect has not been studied. Tolerogenic DC may directly block antibody production through inhibition of BlyS and APRIL, factors that DC use to directly induce immunoglobulin production and class switching in B cells [19]. Alternatively, tolerogenic DC may indirectly prevent antibody production through the inhibition of T-cell helper function.

**Figure 5:**
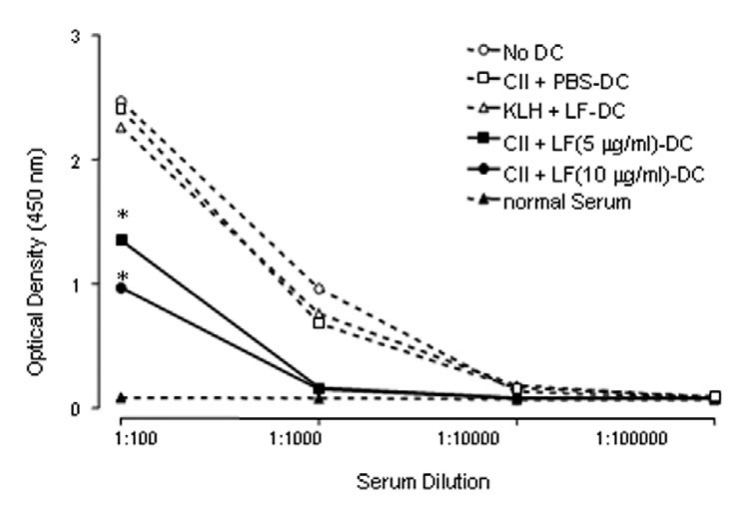
Inhibition of CII-specific antibody production in arthritis mice with LF-treated dendritic cells. Blood was taken 40 days after arthritis onset and serum levels of anti-type II collagen (anti-CII) immunoglobulin were determined using sandwich ELISA. Results show average levels of antibody expressed as the optical density for experimental and control groups (*n *= 6 per group/experiment). *P *< 0.05 versus the control DC-treated group. KLH, keyhole limpet hemocyanin.

In order to assess whether LF-treated DC pulsed with CII actually inhibit CII-specific antibody responses, we evaluated the serum levels of anti-CII immunoglobulin in DBA/1 mice 37 days following the arthritis onset. Using the same protocol as for induction of CIA, we used mice receiving LF-treated DC pulsed with CII, mice receiving LF-treated DC pulsed with PBS, mice receiving PBS-treated DC pulsed with CII, and mice receiving PBS-treated DC pulsed with PBS. A high titer of anti-CII antibody was seen in control DC pulsed with CII (Figure 5). Administration of LF-treated DC pulsed with CII resulted in a marked decrease in antibody production, although there was no essential difference between the two concentrations of LF used on the DC (Figure 5).

The control for this experiment omitted DC immunization, in which there was no inhibition of antibody production as compared with animals that received CII-pulsed DC without LF treatment. This suggests that CIA is not augmented by CII-pulsed DC, but instead that the CII-pulsed LF-treated DC actually inhibit the initiated autoimmune process.

### Histological assessment

**Figure 6:**
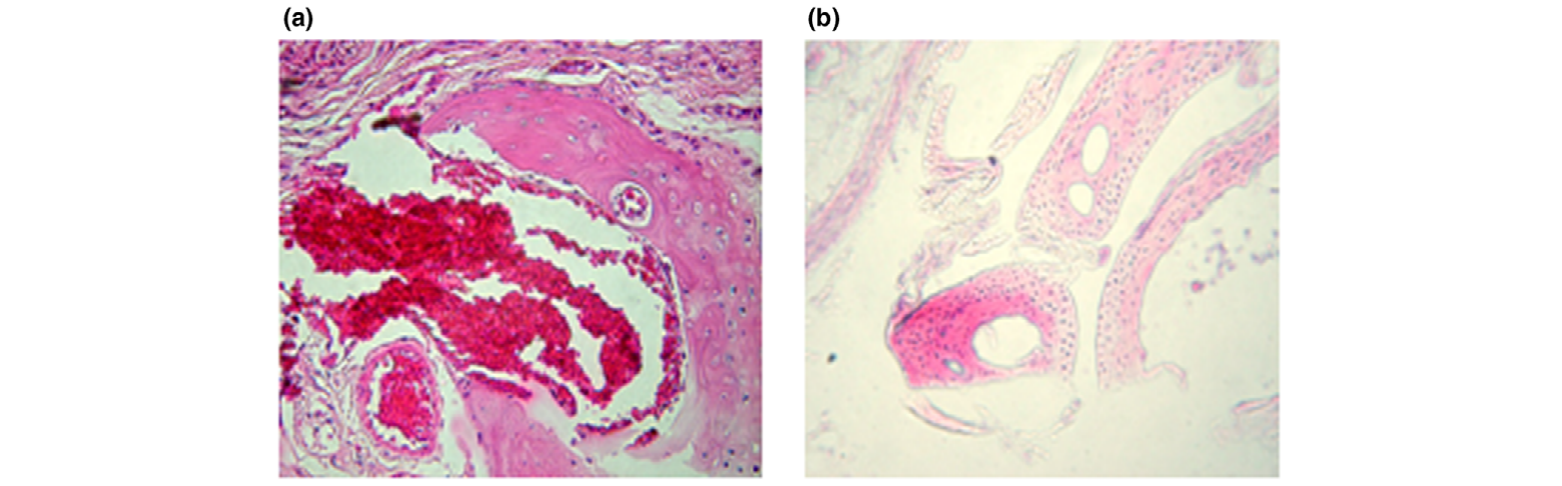
Histological joint sections from arthritic mice with CII-pulsed treated dendritic cells. H & E-stained sagittal sections of proximal interphalangeal joints from collagen-induced arthritis mice. (**a**) Control mouse shows severe edema, congestion, and monocyte infiltration; the bone surface became uneven. (**b**) The majority of joints from mice injected with LF 15-0195-treated dendritic cells have normal morphology with a smooth articulation cartilage surface, and an absence of inflammatory cell infiltrate and edema. Original magnification × 100.

Although we have demonstrated a clear inhibition of arthritis manifestation using the average arthritis score per affected paw, we further sought to examine histological differences induced by treatment with the CII-pulsed LF-treated DC. Animals injected with LF-treated DC, or control animals, were therefore sacrificed 37 days after arthritis onset and their joints were examined in serial sections. We observed that control DC-treated mice exhibited severe synovitis, pannus formation, and bone erosion (Figure 6a). A marked mononuclear cell infiltration was also observed. In contrast, the joint histology of the mice injected with LF-treated DC revealed markedly attenuated morphological changes, cellular infiltration, and the preservation of normal-appearing cartilage (Figure 6b). The histological verification of the arthritis score (Table 1) strongly suggests that the CII-pulsed LF-treated DC are a potent tolerogenic agent that is useful for inhibition of T-cell-mediated autoimmune responses.

## Discussion

The utilization of DC as adjuvants for vaccination has been well described in the literature [20-22]. This is due to the fact that mature DC are recognized as the most potent antigen-presenting cells. It is also well known, however, that immature DC can act as tolerogenic DC and are also potent inducers of tolerance in an antigen-specific manner [23,24]. Attempts have been made to prevent autoimmune diseases through the use of DC-based vaccination [25-27]. Unfortunately, the advances of the understanding of DC vaccine have not been paralleled by development of a means of actually inducing tolerance to the autoantigens.

The use of immature DC as therapeutic tools has had limited success in the treatment of autoimmune diseases. One reason preventing DC-based tolerance is the fact that, once immature DC are introduced into the host, a maturation event may occur that would actually cause immunogenicity instead of tolerance [4,5]. Nevertheless, investigators have attempted to generate such 'tolerogenic DC' using alterations in culture conditions, including low-dose GM-CSF in culture [28], the addition of inhibitory cytokines (IL-10 or IL-4) [29,30], or crosslinking of such DC suppressive surface molecules as the CD200 receptor [31].

A more direct method of targeting DC maturation involves blocking signal transduction pathways that are necessary for the DC to differentiate. A pathway known to be involved in DC maturation is the cascade that leads to activation of the transcription factor NF-κB. Zanetti and colleagues established that the RelB component of NF-κB is critical for DC maturation *in vivo *[6]. Through ablating the RelB gene, they showed a lack of mature DC *in vivo*, as well as immune hyporesponsiveness [6]. The demonstration that immature DC from RelB knockout mice were actually tolerogenic was made through experiments in which DC from RelB knockout animals were pulsed with KLH and used to immunize mice. This resulted in an antigen-specific hyporesponsiveness to KLH that was transferable through a T-regulatory-like cell [32].

The blockade of NF-κB activation has been used therapeutically to generate immature DC by Saemann and colleagues [33] using the thiol antioxidant pyrolidine dithiocarbamate. These DC were able to inhibit alloreactive T-cell responses, as demonstrated by a reduced ability to stimulate a MLR. Another method of suppressing NF-κB activity is through chemical blockade of proteasomes. The proteasome inhibitor PSI, a low molecular inhibitor of IκB-degrading proteasomes, was used to induce the *in vitro *generation of immature DC. These DC were unable to stimulate a MLR and caused a Th1 to Th2 shift in cytokine production [34]. Unfortunately, pyrolidine dithiocarbamate and PSI are both associated with nonspecific suppressive effects on other cellular metabolism pathways, and have not been used for clinical purposes. In this study, we generated a type of tolerogenic DC using the selective IKK/NF-κB inhibitor, LF, for applications as a tolerogenic agent. LF-treated DC exhibited potent tolerogenic properties, which inhibit specific autoimmune responses.

Other inhibitors of DC maturation have been described to inhibit activation of NF-κB directly or indirectly. Among such inhibiting agents are curcumin [35], ganglioside GD1a [36], dexamethasone [37], vascular endothelial growth factor [38], *n*-acetylcysteine [39], and aspirin [40]. Conversely, agents that induce DC maturation – such as TLR-7 agonists [41], TRANCE [42,43], tumor necrosis factor and its related homolog LIGHT [44] – are also known to activate NF-κB. Based on the critical importance of this pathway on DC maturation, *ex vivo *inhibition of NF-κB on DC has been performed using decoy oligonucleotides for the prevention of transplant rejection in liver [45] and cardiac models [46]. Unfortunately, although immune modulation was observed, the effects were not clinically significant.

The immunopathogenesis of RA pathology is complex and incompletely understood. There is strong evidence to implicate MHC class II as an important marker of genetic susceptibility to RA, which implicates T cell-antigen-presenting cell interaction in a fundamental way in the initiation and perpetuation of the autoimmune process. Indeed, the synovitis of RA is characterized by extensive T-cell activation [47]. Clinical efficacy of immune modulating agents, such as methotrexate [48] and infliximab [49], implicates chronic inflammation being secondary to an immune-mediated process. Indeed, successful T-cell-based therapies such as inhibition of costimulation by CTLA4 have recently been reported. Current concepts suggest that synovitis in RA is the result of increased autoreactive effector cell activity and the corresponding decrease in immune regulatory cell function. Furthermore, clinically effective treatments, such as infliximab [50] and autoantigenic vaccination [51], are associated with increased numbers of regulatory T cells in the periphery. Animal models of RA have attempted to recapitulate key elements of RA, although none has done so with complete fidelity. For example, in experimental models the transfer of regulatory cells can prevent arthritis [52], while the depletion of said cells results in accelerated disease [53]. On the basis of the link between immune regulation and remission of RA pathology, we decided to explore the use of LF as an immune modulator in this system.

In order to determine the possible clinical relevance of such LF-treated DC for inducing antigen-specific tolerance or hyporesponsiveness, we assessed their ability to modulate disease progression in the murine CIA, as an experimental model of RA. CIA mirrors many aspects of RA in terms of cellular and immune responses, and has been extensively used to screen therapeutic agents in RA. There are, however, several aspects in which the processes differ. The formation of anti-CCP antibodies and rheumatoid factors is the serological signature of RA, but these autoantibodies are absent from CIA. We chose to examine CIA as a well-defined model of autoimmune arthritis that allows an examination of the role of host immune response to an autoantigen, in this case CII. Our experimental protocols consisted of administering CII-pulsed LF-treated DC on day 12 following the CII priming of animals. This delayed administration of the LF-treated DC was performed to assess whether there was inhibition of an already established immune response. We observed a decrease in the mean clinical score per affected paw in the mice injected with LF-treated DC, compared with control DC. At day 11 after arthritis onset, there was a fivefold difference between the control DC and the LF-treated DC groups in terms of clinical score. Differences in the clinical scores between the control DC and LF-treated DC groups were maintained for the length of the experimental observation, which was 37 days after the arthritis onset. Dutartre's group previously reported that systemic LF administration to CIA mice inhibited development of arthritis but did not modify the Th1/Th2 balance, inducing a switch towards Th2 for preventing arthritis [18]. Owing to some concern regarding the *in vivo *toxicity of LF, however, which has been previously reported [7], herein we used an alternative approach to generate tolerogenic DC by *in vitro *treatment with LF. In addition, *in vitro *treatment of the DC with LF may allow exposure of DC to higher concentrations than would be available *in vivo*.

This study serves as a foundation for establishing parameters for the generation of an antigen-specific tolerogenic treatment approach using LF-treated DC. This is the first demonstration that *in vitro*-generated antigen-specific immature DC may be used as a tolerogenic vaccine for the treatment of autoimmune arthritis.

## Abbreviations

CIA = collagen-induced arthritis; CII = type II collagen; DC = dendritic cells; ELISA = enzyme-linked immunosorbent assay; FCS = fetal calf serum; GM-CSF = granulocyte-macrophage colony-stimulating factor; H & E = hematoxylin and eosin; IKK = IκB kinase; IL = interleukin; KLH = keyhole limpet hemocyanin; LF = LF 15-0195; LPS = lipopolysaccharide; mAb = monoclonal antibody; MHC = major histocompatibility complex; MLR = mixed leukocyte reaction; PBS = phosphate-buffered saline; RA = rheumatoid arthritis; Th = T helper cell; TNFα = tumor necrosis factor alpha.

## Competing interests

The authors declare that they have no competing interests.

## Authors' contributions

IP carried out the CIA studies and the *in vivo *immune assays, and drafted the manuscript. ML carried out *in vitro *and *in vivo *immune assays. XfZ, XsZ, and TEI participated in the CIA assessment. HS and BG performed the pathology examinations. TEI, BF, MS, and CV helped to draft the manuscript. RZ, GS, RDI, and W-PM participated in the study design and coordination, and helped to draft the manuscript. All authors read and approved the final manuscript.

## Figures and Tables

**Table 1:** Joint pathology score

Group	Score	Mean ± SEM	*P *value
Control dendritic cells	3, 3, 4, 3, 3.5, 3	3.250 ± 0.171	
LF 15-0195-treated dendritic cells	1, 0, 1.5, 1, 1.5, 1	1.000 ± 0.224	0.000283

Histopathologic changes are scored using the following parameters. Synovial inflammation (infiltration and hyperplasia) is scored on a scale from 0 to 4, depending on the amount of inflammatory cells in the synovial cavity (exudates) and synovial tissues (infiltrate). Each joint is scored separately by two individuals unaware of the treatment protocol, using the following scale: no inflammation = 0; slight thickening of lining layer and/or some infiltrating cells in the sublining layer = 1–2; thickening of lining layer and/or a more pronounced influx of cells in the sublining layer = 3; and presence of cells in the synovial space, thickening of lining layer, and synovium highly infiltrated with numerous inflammatory cells = 4.
